# A qualitative non-participant observational study of non-prescription counseling in community pharmacies

**DOI:** 10.1016/j.rcsop.2025.100611

**Published:** 2025-05-03

**Authors:** Helene Marie Haldorsen Gombos, Tonje Krogstad, Marthe Rambøl Bjørknes, Hege Sletvold, Milada Cvancarova Hagen, Karin Svensberg

**Affiliations:** a Pharmacy, Department of Life Sciences and Health, Faculty of Health Sciences, Oslo Metropolitan University, OsloMet – storbyuniversitetet, Postboks 4, St. Olavs plass, 0133 Oslo, Norway; b Faculty of Nursing and Health Sciences, Nord University, Postboks 324, 7501 Stjørdal, Norway; c Department of Nursing and Health Promotion, Faculty of Health Sciences, Oslo Metropolitan University, OsloMet – Storbyuniversitetet, Postboks 4, St. Olavs plass, 0133 Oslo, Norway; d Department of Pharmacy, Uppsala University, Box 580, 751 23 Uppsala, Sweden

**Keywords:** Pharmacy, Community pharmacy, Non-prescription products, OTC medication, Selfcare, Self-treatment, Counseling, Communication, Observation, Qualitative research, Norway

## Abstract

**Background:**

The global rise in self-care and non-prescription product sales allows more people to self-treat minor ailments, and pharmacies have a key role in guiding this use. However, discrepancies between counseling standards and practice suggest a gap in ensuring safe and informed self-medication, necessitating exploration and understanding.

**Objectives:**

This study investigates counseling on all non-prescription products in pharmacies, with the following objectives: 1) assess compliance with The Norwegian OTC Counseling Standard (the Standard), 2) identify factors predicting standard compliance, and 3) describe the content of non-prescription counseling.

**Methods:**

A non-participant observation study (*n* = 275) was conducted in the self-care section of Norwegian pharmacies (*n* = 7) from September 2022 to March 2023. During the encounters, 494 products were purchased. Notes from the observations were transformed into transcripts. The transcripts were analyzed using Content Analysis, where codes were quantified and compiled into a spreadsheet. Statistical analysis was performed using Chi-square tests and Logistic regression to evaluate standard compliance and predictive factors.

**Results:**

Full compliance with the standard was 14.6 %; however, ∼40 % of product inquiries did not meet the requirements. Busier pharmacies showed higher standard compliance compared to quieter, and counseling starting at the shelves was more comprehensive. Moreover, pharmacists were likelier than pharmacy technicians to provide information about OTC products (*p*-value = 0.01). Standard compliance was higher for other non-prescription products than for OTC products. Assessment of the customer's product needs typically revolved around previous use, the intended user, and symptoms, with less emphasis on patient-specific factors (e.g., contraindications, interactions). Information was usually practical, for example, instructions on use and dosage, while discussions on effects and adverse drug reactions depended on customer prompts.

**Conclusion:**

The study found infrequent compliance with the standard, with information often being generic and not tailored to customer needs. OTCs have lower standard compliance than other products sold in pharmacies. Further efforts need to be made to improve counseling, including revising the standard focusing on communication skill promotion and expanding to consider all health-related products in the self-care section. This could improve self-treatment and reduce the burden on other healthcare providers.

## 1 Introduction

The sales of over-the-counter (OTC) medications and dietary supplements are increasing worldwide, with more self-treating of minor ailments.^1^^,^^2^ In 2022, 8.5 billion packages of OTCs and 1.3 billion packages of minerals and vitamins were sold in Europe.^3^ The World Health Organization (WHO) defines self-care as “what people do for themselves to establish and maintain health, prevent and deal with illness.”^4^^,^^5^ This includes maintaining a healthy diet, exercising, and taking dietary supplements and medications to improve and restore good health. Governments are investing in self-care programs to alleviate strain on the healthcare sector, and pharmacies may contribute to this effort.^6^, ^7^, ^8^, ^9^ Counseling on non-prescription products (NPPs) to ensure their correct use and increase health literacy has been introduced as a key solution for better self-care. Prompting self-care for minor ailments empowers patients to actively participate in their health and may decrease physician visits, thereby lessening the burden on the healthcare system. Products sold in pharmacies to promote self-care include OTCs, dietary supplements, herbal drugs, and medical technical equipment (MTE). In this article, they are collectively defined as NPPs. Reclassifying previously prescription-only medications as OTCs (OTC switch) has made them more accessible to the public.^10^, ^11^, ^12^, ^13^ However, inappropriate use also occurs alongside the widespread use of NPPs. This includes excessive use, incorrect use, and use for the wrong indication.^10^, ^11^, ^12^ Many customers perceive OTCs as harmless because they do not require a prescription, assuming that all potentially harmful medications are regulated through prescriptions.^14^^,^^15^ By ensuring that information on both benefits and risks is made available to them, customers may be better equipped to make self-care decisions.

Pharmacies often serve as a primary source for customers seeking NPPs and play an integral role in improving medication use by helping them find the right product for self-care.^5^^,^^16^^,^^17^ Many other sources of information about NPPs are available to customers today, with varying degrees of reliability and validity. For example, traditional and social media contain advertisements, articles, and recommendations for self-care. Healthcare resources are also accessible through a simple online search. Many people share their experiences or opinions in online comment sections,^18^ and customers may rely on the experiences of relatives and friends.^19^ In this context, pharmacy employees can supplement information from other sources and correct misunderstandings. According to Bergmo et al., healthcare providers and government web pages are perceived as the most reliable sources of health information by the public.^20^ To avoid incorrect medication use, customers need adequate knowledge, while pharmacy employees also need the knowledge and skills to assess the customer's information needs.

Pharmacies' tasks include ensuring that medications, including OTC, are used safely and effectively by, for example, counseling on their use and helping patients choose the correct medication. In Norway, this responsibility is regulated by law. The law states that the pharmacy must ensure that the customer has sufficient information to use the product correctly, as well as information about harmful effects if there is reason to suspect misuse.^21^ To ensure good practices regarding OTC counseling by pharmacies, standards or guidelines have been developed in several countries22., 23., 24., 25. A standard can help ensure consistent quality in every customer interaction but may limit the patient-centered counseling approach. For the standard to be valuable, it must be used actively, evaluated, and developed continuously. Several studies have assessed the level of OTC counseling by pharmacy personnel.^16^^,^^26^, ^27^, ^28^, ^29^, ^30^, ^31^, ^32^, ^33^, ^34^, ^35^, ^36^, ^37^, ^38^, ^39^ The two most common methods used to assess OTC counseling without a self-reported bias are the simulated patient method and non-participant observational studies. The simulated patient method uses predefined scenarios to determine adherence to therapeutic-specific guidelines. Observational studies have been conducted to evaluate overall standards, albeit to a lesser extent.^26^, ^27^, ^28^ Regardless of the method used, current studies suggest significant room for improvement in pharmacy OTC counseling practices. Pharmacy employees generally provide better counseling when customers present with symptoms rather than asking for a specific product.^16^^,^^26^^,^^27^^,^^35^^,^^37^^,^^40^^,^^41^ Studies also show discrepancies regarding the difference between pharmacists and pharmacy technicians, with some finding that pharmacists provide more information^32^^,^^36^^,^^37^ and others reporting no significant difference^35^ or that pharmacy technicians offer more.^16^ Some employees report difficulty engaging customers they perceive as uninterested in conversation.^41^ Further, customers are often found to be confident in self-diagnosing^40^ and do not need counseling if they have previous experience with the product.^42^

The Norwegian Pharmacy Association has established an overreaching standard for OTC counseling in Norway. (Box 1) The extent to which the Norwegian OTC Counseling standard (the Standard) has been implemented has yet to be investigated. Research in other Nordic countries is limited and has shown room for improvement in counseling on OTC products.^38^^,^^39^ Likewise, previous research into standard compliance focuses on OTC guidelines and has not investigated needs assessment and information provision for other NPPs.^27^^,^^37^

## 2 Aim

The goal of this study is to investigate the implementation of the Standard, with a focus on describing the non-prescription counseling process by addressing the following questions:

1. To what extent do employees comply with the OTC medication counseling standard, specifically regarding unsolicited information provision and assessment of customers' needs and information needs?
2. Which of the selected customer and employee factors are associated with standard compliance regarding assessment, information provision, and information provision according to the customer's needs?
3. What topics are discussed during assessments, and what information is given to customers during the NPP-related encounters?

## 3 Methods

### 3.1 Study design and observation method

Non-participant observations were performed in the self-care section in pharmacies.^43^ The study is reported in accordance with the SRQR guidelines.^44^

### 3.2 Study context

Box 1 provides key details about NPP practices, the Standard, and employee roles in Norwegian pharmacies.^45^ OTC products are available on pharmacies' shelves, allowing customers to find and select those they wish to purchase. Most pharmacies have separate sale points for NPPs in the self-help section, but these products may also be bought at the prescription counter.

Box 1 **Selected key details regarding NPP practices, the Standard, and employee roles in Norwegian pharmacies.21,22,45,46**

**Table:** 

NPP – non-prescription products	
•	Medication is classified as prescription only or non-prescription. One product (Viagra) is classified as non-prescription requiring pharmacist counseling, and one product (Duraphat toothpaste) is classified as non-prescription with counseling (pharmacist or pharmacy technician)
•	Other NPPs sold in pharmacies include MTE (medical technical equipment), dietary supplements and other products to treat or prevent disease
•	OTC (Over-the-Counter) medication on the LUA (Legemidler utenom apotek - medication available outside pharmacies) list may be sold in grocery or convenience stores. Employees in these stores are not permitted to counsel on these OTC medications.
•	Dietary supplements and other non-regulated products are sold in various locations, such as grocery stores, online and in specialized herbal healthcare stores.
*The Standard*	
•	Information about use should be easily available to the customer
•	Information and advice should be provided unsolicited (1 – *Unsolicited information provision*).*
•	The customer's needs should be assessed (2 – *Assessment of customer's needs*). *
•	Counseling should be adjusted accordingly (3 – *Assessment of customer's information needs*).*
•	Discretion should be maintained during counseling and sales.
•	Applies to all employees
•	Only applies to OTCs not other NPPs
*Pharmacies and employee roles*	
•	Most pharmacies are chain-owned. The pharmacy owner does not have to be a pharmacist.
•	Pharmacists have either a bachelor's or a master's degree. They work independently both in prescription and non-prescription sales. Pharmacists are authorized healthcare personnel. The pharmacy manager must hold a master’ s degree.
•	Pharmacy technicians possess a high school diploma (authorized healthcare personnel). They require oversight from a pharmacist when filling a prescription but can work independently in the self-care section. There is no legislation defining the level of counseling that a pharmacy technician may provide, and guidance is sought from a pharmacist is at the pharmacy technician's discretion. The self-care section is often staffed by pharmacy technicians.
•	Pharmacy aids are students or other employees who have no formal education in pharmacy or are yet to complete their studies. They should work under supervision, but there is no legislation defining how independently they can work in the self-care section.
	*Sections of the Standard that has been used to inform the outcomes of the study are indicated with a number that corresponds to the outcome number.

Alt-text: Box 1

### 3.3 Study setting, recruitment, and sample

The sampling goal was to recruit 1–3 pharmacies per pharmacy chain and 1 standalone pharmacy. All community pharmacies in the Oslo metropolitan area that belonged to the 3 pharmacy chains were invited to participate in the study. This was done through an e-mail distributed by the chains, encouraging pharmacy managers to participate in the observations. Only 1 pharmacy manager responded to this invitation. As a result, additional pharmacies were recruited by directly contacting the pharmacy managers. Eight pharmacies were approached in this manner. Two of these pharmacies declined to participate. In total, 7 pharmacies participated, representing all major pharmacy chains (Pharmacy Chain 1, *n* = 3; Pharmacy Chain 2, *n* = 2; and Pharmacy Chain 3, *n* = 1) and 1 standalone pharmacy. These pharmacies were located in various areas, from local pharmacies in shopping streets to smaller shopping centers and larger shopping malls. Moreover, the pharmacies varied in size (number of employees, daily encounters, and busyness).

All available pharmacists and pharmacy technicians in each pharmacy were informed about the scope and theme of the study and what their participation entailed. No one declined to participate. Customers were recruited while waiting in the self-care section and were observed throughout the encounter. They were approached before making contact with an employee. The only exclusion criteria during the observations were the inability to contact the customer before employee contact and the customer drawing a queue number for prescription pick-up. Customers who appeared to be under 18 were excluded, but no such customer was encountered. All other available customers were approached, provided the observers were not already observing a different customer. A predefined sample size was not set due to the qualitative data collection method, but around 50 observations per pharmacy were aimed for. Observations in the final two pharmacies were completed before reaching 50 observations due to a lower customer turnout.

### 3.4 Data collection

Observations were conducted between September 2022 and March 2023. They were carried out on weekdays at various times throughout the day to account for customer flows. The first pharmacy was used as a pilot, with audio recordings of the encounters. However, the method was adjusted due to poor recording quality and recruitment difficulties. Instead of recordings, observation notes were written down using 2 predefined forms (Appendix 1). One form included space for free text, where the conversations were noted as close to verbatim as possible, and the other included checkboxes and a note field. This allowed the observers to gather detailed descriptions. Two observers conducted simultaneous, independent observations using one of the two forms each. The observers positioned themselves strategically to observe and hear as much as possible while maintaining enough distance to avoid disturbing the encounter. Since the counseling could occur at any point in the pharmacy, the positions of the observers were not fixed. The notes were discussed and unified immediately after observations to capture as much detail as possible. Three observers (HG, TK, MRB) were involved, and HG was present at all observations. In the last 2 pharmacies, HG made the observations alone after gaining considerable experience from previous observations. The information gathered in the forms was transformed into detailed descriptions of each encounter, including the verbatim conversations. The pilot recordings were transcribed and included in the data sample.

Each employee was assigned a code consisting of P for pharmacist or T for pharmacy technician, followed by a number. The total number of male and female employees in each pharmacy was recorded, but the gender of the employee involved in each encounter was not noted to prevent possible identification. The only characteristics noted about customers were gender and age group. Some observations continued to the prescription area, but no information was reported about prescription sales.

### 3.5 Collected variables and definitions

The collected data material encompassed all observed encounters. However, for the statistical analyses, encounters where the customer left without interacting with an employee and where only non-medical products (e.g., cosmetics, toothbrushes) were sold were excluded. Non-medical products were included when bought together with other products. One encounter was excluded because only 1 out of the 2 customers in the encounter consented to the observation. Statistical analyses were conducted at the product level, not the encounter level, as many customers bought more than 1 product, and the information exchange differed within the encounter.

The detailed descriptions of the encounters were imported to NVivo (release 1.7.1). An analysis inspired by Content analysis was performed.^47^ All data were coded using a text-driven inductive approach, and the codebook was created after the data set was completely coded. After initial coding by coder HG, the codes were discussed between the original coder (HG) and a second coder (TK). The code book (see Appendix 2 for an example) with instructions was then shared with 2 additional coders (HS and KS), who coded a selection of the observations. The codes were discussed to ensure validity in the coding process. The codes were further quantified, compiled into a spreadsheet, and used to address the aim of this study. The main outcomes are described in Table 1, and the collected variables in Table 2.

**Table 1:** Main outcomes. The outcomes were defined and dichotomized using the Standard.

		“Yes”	“No” ⁎
Outcome 1:	*Unsolicited information provision*	Information provided about the product. The word “unsolicited” was not emphasized, and “Yes” was assigned even if the customer asked for the information, (i.e., information was provided but not unsolicited).	No information was given or information on only price and/or package size without further other information.
Outcome 2:	*Assessment of customer's needs*	The employee asked about the customers medical needs, or the customer provided this information unprompted.	No questions about needs where asked. “Have you used this previously?” without any follow-up questions
Outcome 3:	*Assessment of customer's information needs*	The employee asked questions about the customer’ s needs and tailored the counseling according to the conversation with the customer.	Information was given but it was generic and not based on information gathered from the customer.

⁎ NA was used for products where assessment or information was not deemed necessary (e.g., face masks, urine sample containers, fluoride mouthwash, saline solutions, and cough drops without medicinal substances).

**Table 2:** Predictive factors selected to evaluate their impact on NPP counseling. Predictive factors were selected through discussions within the research team and inspired by factors identified in a similar study.^27^

Predictive factor	Values	Explanation
Approximate age of the customer	18–49 years vs. 50–69 years vs. 70+ years	Evaluated and agreed on by the observers. The customer was not asked.
Gender of the customer	Male vs. female vs. non-binary	Evaluated and agreed on by the observers. The customer was not asked.
Specific product requests	Yes vs. no	“Yes” was assigned if customers asked for specific products or found products themselves.
The product group purchased	*OTC* vs. *dietary supplements* vs. *other medical product* vs. *non-medical product* vs. *no sale*	Defined using the online catalog of the pharmacy chains. Other medical products include MTE, diagnostic equipment, and other products used for a medical condition. Non-medical products include beauty products or other products sold in the pharmacy that are not used for medical purposes.
Previous physician contact	Yes vs. no	“Yes” was assigned if the customer reported previous physician contact voluntarily or after being questioned. “No” was assigned if the customer answered no or reported not having spoken with a physician. NA was assigned if the topic was not addressed.
Employee role	Pharmacist vs. pharmacy technician	No distinction was made between pharmacists with master's and bachelor's degrees; pharmacy aids were included with pharmacy technicians because of low numbers and to avoid identification.
Busyness of the pharmacy	Quiet vs. moderately busy vs. busy	Busyness was based on how busy the observers found the pharmacy during the observation; it was adjusted for the typical customer flow in the pharmacies, as some pharmacies were observed around school holidays and were quieter than usual. 1 pharmacy was classified as busy, 3 as medium busy, and 3 as quiet.
Starting location of counseling	At shelf vs. at point of sales	

NPP: non-prescription products; OTC: Over-the-counter medication; MTE: Medical technical equipment.

Topics of assessment and information.

The quantified codes were depicted in bar graphs using Microsoft Excel for Mac (16.92).

### 3.6 Statistical methods

Frequency data were extracted from NVivo. All analyses were conducted using SPSS (ver. 29). Categorical variables are presented descriptively as frequencies and percentages, and continuous variables are presented as medians with min-max values because the sample size was limited and the variables had skewed distribution. Crude associations between the outcomes (“unsolicited information provision,” “assessment of customer’ s needs,” and “assessment of customer’ s information needs”) and a selected potential predictive factor (“product group”) were assessed with a chi-square test. This predictive factor was chosen because the Standard and previous research have focused on OTC and to show how counseling differed between different product groups. Three multiple logistic regression models were fitted to identify the possible predictive factors associated with the outcomes. The first model included covariates regarding the customer (age, gender, specific product request, and product group), the second model included variables related to the employee (education, pharmacy busyness, and location), and the third model included all variables considered to be associated with standard compliance. The three models showed the same factors influencing standard compliance; therefore, the full model was chosen to present the data. The results from the logistic regression model were presented as odds ratios (ORs) with 95 % confidence intervals (CIs). The CIs were constructed using bootstrapping to provide more robust lower and upper bounds for the estimates. The models were created for all product groups, even though the Standard does not cover other NPPs. To address this, sensitivity analyses were conducted to evaluate the impact of OTCs on the results, focusing exclusively on OTCs. In these analyses, the model was adjusted for relevant variables when fitting all variables was not feasible. All analyses were considered exploratory, so multiple testing was not corrected for, and *p*-values <0.05 were considered statistically significant.

### 3.7 Ethics

As no identifiable data were collected, and the research was not categorized as healthcare research,^48^ approval from the REK (Regional) was deemed unnecessary. The study and data management were presented to and endorsed by SIKT (the Norwegian Agency of Shared Services in Education and Research). The study was conducted per the principles of the Declaration of Helsinki. Participation was voluntary for both employees and customers. Customers were approached before contact with an employee and asked to give oral consent for the observation. Customers participating in the audio-recorded pilot signed informed written consent. Pharmacy employees signed informed consent forms before participating in the observations.

## 4 Results

### 4.1 Sample characteristics

**Fig. 1:**
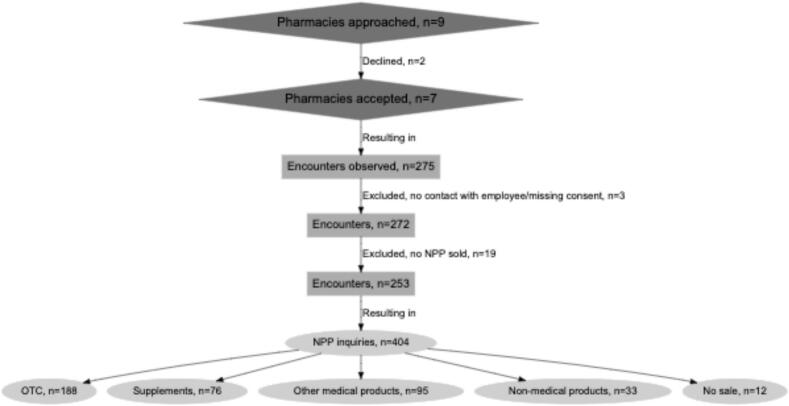
Flow chart depicting the inclusion and exclusion of pharmacies, encounters, the resulting number of NPP inquiries, and the different product groups. NPP: Non-prescription products; OTC: Over-the-counter medications.

In total, 275 encounters were observed in 7 pharmacies, with a median of 38 observations each (min-max 16–54). In total, 24 pharmacists (*n* = 4 [16.7 %] male) and 28 pharmacy technicians (*n* = 0 male) participated. Fig. 1 describes the recruitment of pharmacies, the number of encounters, and product inquiries.

**Fig. 2:**
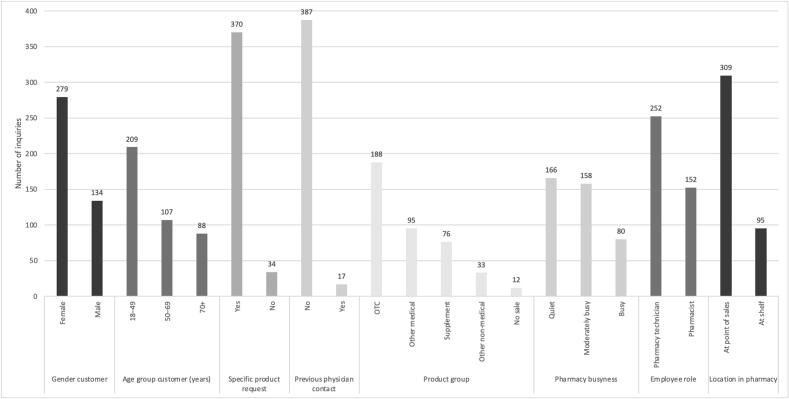
Distribution of the 404 NPP (Non-prescription products) inquiries across the possible predictive factors. OTC: over-the-counter medication.

Fig. 2 describes the distribution of the inquiries across the possible predictive factors. The customers were predominantly female (69 %) and in the 18–49 age group (52 %). Most customers (92 %) requested a specific product at the pharmacy and had not been in contact with a physician (98 %). There were more sales of other non-prescription products (*n* = 204) than of OTC (*n* = 188). The pharmacists conducted 91 encounters, while pharmacy technicians were involved in 162 encounters.

Overall standard compliance.

In 14.6 % (*n* = 59) of the product sales, the employee met all 3 requirements (assessment of customer's needs, unsolicited information provision, and assessment of customer's information needs). Two out of 3 requirements were met in 14.4 % (*n* = 58) of the sales, and only 1 requirement was met in 25.2 % (*n* = 102). Of the product sales evaluated, 7.7 % (*n* = 31) were deemed not to require counseling (for instance, only saline was sold). None of the requirements were met in 38.1 % (*n* = 154) of sales.

**Fig. 3:**
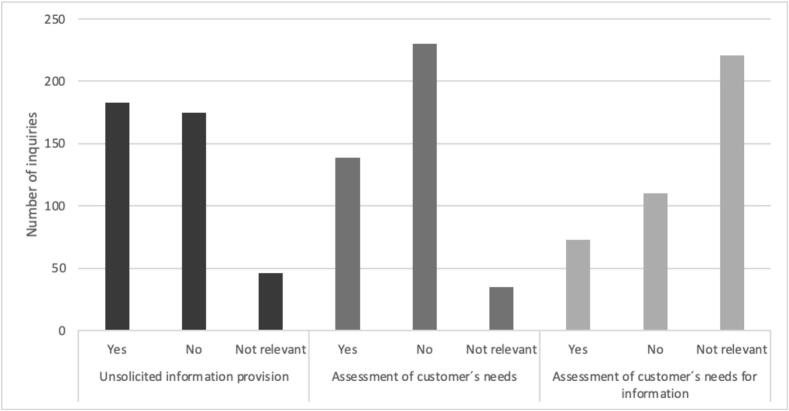
The frequency of compliance with the Standard for providing unsolicited information, assessing customer needs, and fulfilling information requirements.

Fig. 3 describes how often each of the requirements was met.

### 4.2 Outcome 1: unsolicited information provision

Information was provided for half of the assessed products (*n* = 183 of 358; Fig. 3). At the product group level, information was provided for 53.7 % (*n* = 101 of 188) of the product inquiries about OTCs, 53.8 % (*n* = 43 of 80) for other medical products, and 44.0 % (*n* = 33 of 75) for dietary supplements. Information was also provided for other non-medical (n = 3 of 6) and no sale (n = 3 of 9); percentages are not presented due to the low number of cases.

The odds of standard compliance on information provided were almost 6 times higher if the customer's needs were assessed compared to when no assessment was made (Table 3). When the conversation started at the point of sales, information was almost 60 % less likely to be provided compared to when the conversation started at the shelf. For moderately busy and busy pharmacies, information was around twice and almost 2 times more likely to be provided, respectively, compared to quiet pharmacies (Table 3). The main results were confirmed with sensitivity analyses. When considering only OTC products, the same factors predicting standard compliance were statistically significant. In addition, if the employee was a pharmacist, the odds of information being provided were almost three times higher compared to when a customer was met by a pharmacy technician (OR = 2.96, 95 % CI = [1.18–7.29]; Supplementary file 1).

**Table 3:** Factors associated with unsolicited information provision, assessment of customer’ s needs, and assessment of customers information needs on NPPs in Norwegian pharmacies.

Variables	OR	95 % CI	*p*-value
**Outcome 1:***Unsolicited information provision*			
**Assessment**			
*No (reference)*	1		
*Yes*	5.89	3.25–12.897	0.001
**Starting location of counseling**			
*At shelf (reference)*	1		
*At point of sales*	0.43	0.22–0.73	0.006
**Product group**			
*OTC (reference)*	1		
*Dietary supplement*	0.50	0.29–0.79	0.016
*Other medical product*	0.63	0.32–1.098	0.140
*Other non-medical product*	0.84	⁎	0.730
*No sale*	0.14	0.00–1.498	0.006
**Pharmacy busyness**			
*Quiet (reference)*	1		
*Moderately busy*	2.22	1.21–4.58	0.007
*Busy*	2.98	1.43–7.55	0.001
**Outcome 2:***Assessment of customer’ s needs*			
**Customer gender**			
*Male (reference)*	1		
*Female*	2.26	1.39–4.25	0.003
**Specific product**			
*Yes (reference)*	1		
*No*	8.95	3.68–41.596	<0.001
**Previous physician contact**			
*No (reference)*	1		
*Yes*	3.64	1.22–15.07	0.015
**Pharmacy busyness**			
*Quiet (reference)*	1		
*Moderately busy*	2.51	1.43–4.37	<0.001
*Busy*	2.03	0.98–4.47	0.042
**Starting location**			
*At point of sales (reference)*	1		
*At shelf*	3.58	2.06–6.56	<0.001
**Outcome 3:***Assessment of customer’ s information needs*			
**Assessment**			
*No (reference)*	1		
*Yes*	14.77	⁎	<0.001
**Starting location**			
*At shelf (reference)*	1		
*At point of sales*	0.28	0.09–0.58	0.011
**Product group**			
*OTC (reference)*	1		
*Dietary supplement*	2.33	0.57–10.16	0.162
*Other medical product*	66.26	⁎	<0.001
**Customer age group**			
*18–49 (reference)*	1		
*50–69*	5.00	0.96–52.196	0.014
*70+*	6.06	1.33–70.32	0.005
**Pharmacy busyness**			
*Quiet (reference)*	1		
*Moderately busy*	0.75	0.17–2.98	0.656
*Busy*	7.72	1.87–158.38	<0.001

OR: odds ratio, CI: confidence interval, OTC: over-the-counter medication, NPP: non-prescription product.

Variables that were non-significant were excluded from the models.

⁎ Could not be estimated.

The information most frequently provided is described in Supplementary File 2a. Typically, the information was practical, for example, instructions for using the product (*n* = 41). Information on the length of treatment was often provided for products requiring this (*n* = 36), for example, information on the short-term use of decongestant nasal spray. When describing dosage, employees used different methods of instruction. Some employees explained the dosage quantity and frequency (*n* = 47) or only the maximum total dosage (*n* = 5); others mentioned where to find the dosage (*n* = 15) or just told the customer to “follow the dosage” (*n* = 9) without mentioning the actual dosage. Information about adverse drug reactions was usually only given if the customer asked about them (*n* = 18), and responses from the employees were often deflective.

### 4.3 Outcome 2: assessment of customer's needs

The customer's product needs were assessed in less than 40 % of the product inquiries (*n* = 139 of 369; Fig. 3). Of the 188 OTCs sold, employees assessed the customers' needs in 34 % of the cases (*n* = 64). For other NPPs, the prevalence of assessment was higher, with dietary supplements at 36.8 % (*n* = 28 of 76) and other medical at 43.2 % (*n* = 35 of 81). The incidences of non-medical product-related encounters and those with no sale were insufficient in number for calculation.

Assessment of the customer's needs was almost 9 times more likely if the customer presented with a symptom compared to presenting with a specific product request. When the conversation started at the shelf, the odds of assessment were more than 3 times higher than when it began at the point of sales. The odds of women receiving assessments were more than twice that of men, and the busy pharmacies had more than twice the odds of assessing the customer's needs than the quiet pharmacies (Table 3). The same factors, except customer gender, were statistically significant for OTC products (Supplementary File 1). The most frequent assessments and questions asked during consultations were about the previous use of the product (*n* = 110), who the product was for (*n* = 58), and the customer's symptoms (*n* = 51). These results are described in Supplementary File 2b. The employees rarely addressed issues such as comorbidities, interactions with other medications, pregnancy, and breastfeeding.

### 4.4 **Outcome 3:** assessment of customer's information needs

The information was customized to the customer's needs in 39.9 % (*n* = 73) of the 183 cases where information was given (Fig. 3). Based on the product groups, the information was customized for 18.6 % (*n* = 19 of 102) of the OTC products, 48.5 % (*n* = 16 of 33) of the dietary supplements, 81.4 % (*n* = 35 of 43) of the other medical products, other non-medical (*n* = 2 of 2), and no sales (n = 1 of 3). Percentages are not presented for the last two groups due to the low number of cases.

The likelihood of tailoring information to the customer's needs decreased by approximately 70 % when the conversation started at the point of sales, compared to starting at the shelf. Other medical products had more than 60 times higher odds of the information being according to the customer's needs compared to OTCs. If the customer's needs were assessed, the odds of information being according to their needs were almost 15 times higher than without an assessment. Higher customer age (5 times higher odds for the age group of 50–69 years and 6 times higher odds for the age group of 70+ years) prompted more tailored information compared to lower customer age. Compared to the quiet pharmacies, the busiest pharmacies had almost 8 times higher odds of providing information according to the customer's needs. Because of the low number of cases, the exact size of the effect of each factor is difficult to estimate. However, these factors were highly statistically significant. (Table 3).

A logistic regression model with the same factors for all products could not be fitted for the OTC sensitivity analysis because the statistical power was too low. Therefore, a model with the employee's education level and location in the pharmacy was fitted. This showed that when the conversation started at the shelf, the information was statistically significantly more likely to be given according to the customer's needs (OR = 9.98, 95 % CI = 2.30–69.70; Supplementary File 1).

## 5 Discussion

This study is the first to examine counseling globally on all NPPs sold in pharmacies, using an observational method without pre-established objectives for each customer interaction. Most studies on guideline compliance utilize the simulated patient approach and focus on specific therapeutic or diagnostic guidelines.^30^, ^31^, ^32^, ^33^, ^34^, ^35^, ^36^, ^37^, ^38^ Other non-participant observational studies have assessed guideline compliance using preset criteria, opting not to employ qualitative coding methods during analysis.^27^^,^^29^ These studies focused on OTC medications and omitted other NPPs. Comparing the counseling regarding these different product groups provides a more complete picture of the pharmacy employees as self-care guides. The Standard used in this research pertains explicitly to OTCs, yet the broad nature of the Standard goals allows for its application to other NPPs. It can be recognized that the level of counseling needed for other NPPs may be different than for OTCs. However, as these products may influence the customers' health, awareness of the importance of counseling about this product group is essential. Moreover, assessing the customer's needs and providing information according to their information needs was found to be more frequent for dietary supplements and other medical products than for OTCs. The likelihood of customizing information for other medical products compared to OTCs was substantially higher. Although customized information for dietary supplements did not differ statistically significantly from that of OTCs, it was provided for nearly 50 % of dietary supplement inquiries compared to about 20 % for OTCs. Whether the pharmacy personnel were intentional in this regard was not studied; however, this finding may reflect their unawareness of the scope of the Standard.

To summarize the main findings, pharmacy employees in this study complied with the Standard to some degree. Predictive factors for standard compliance were busy pharmacies, counseling starting at the shelves, and pharmacists' involvement in counseling on OTC medications. Furthermore, NPP counseling typically involved providing generic information seldom tailored to the customer's needs.

The current Standard^22^ outlines the necessary actions without detailing the methods for achieving these goals. On the other hand, standards from different countries are more comprehensive regarding counseling procedures.23., 24., 25. Pharmacy employees in this study rarely met all 3 criteria in the Standard, and none of the requirements were met in 2 out of 5 inquiries. The extent of employees' awareness of the Standard was not studied. Other studies, using both overreaching standards^27^^,^^29^^,^^37^ and therapeutic guidelines,^30^, ^31^, ^32^, ^33^, ^34^, ^35^, ^36^^,^^38^ reported similar results, showing room for improvement in OTC counseling. In some studies, awareness of the available standards was found to be good but was not evident in practice when further investigated.^29^ This indicates that a standard or guideline needs to be designed to be useful in practice, and workers need better awareness of the guideline's contents and how to adhere to them, including ethical conduct. For instance, the statement “advice should be given unsolicited” is unclear since the definition of “unsolicited” may not be easily understood.

Most customers entered the pharmacy with a specific product in mind, and employees seldom questioned its suitability, reflecting respect for customer autonomy or, potentially, a fear of loss of sales.^49^ Previous studies demonstrate similar trends; customers typically receive the requested product without further inquiry, and the employees express that the customers appear uninterested in counseling.^27^^,^^29^^,^^37^ On the other hand, the customers want counseling.^41^ This gap could be reduced by more explicitly showcasing what the counseling could be about.^50^ The most apparent factors determining standard compliance involved employees actively inquiring about the customer's needs and engaging the customers before product selection. Employees tended to ask about previous product use but provided minimal or no additional information if the prior use was confirmed. However, previous use does not guarantee that the customer was adequately informed in the past, nor does it ensure proper future use.^51^^,^^52^ Questions about the intended user of the product and symptoms experienced were common, but inquiries on person-specific factors (e.g., interactions, comorbidities, and pregnancy) were rare. While this study did not evaluate the necessity of additional questions in specific inquiries, the scarcity of such questions is noteworthy. The reasons why the employees in this study did not ask questions are yet to be investigated and should be researched further. In other studies, pharmacists often refrained from asking questions when customers appeared sad, reserved, or in a bad mood.^27^^,^^29^

This study found that information was provided as a generic statement in 3 out of 5 OTC inquiries. How generic information influences the customer's product usage was not investigated. What was shown, however, is that the information provided was practical, with directions for use, dosage, and duration. Regulations dictate that ensuring correct use is essential for pharmacies,^21^ possibly making this the natural focus. Previous research has shown that pharmacists focus on practical information.^53^ In contrast, customers wish to receive information on topics such as adverse drug reactions, interactions, and medication characteristics.^54^ Adverse drug reactions were rarely addressed (*n* = 18) in this study, and only if the customer asked about them. Employees often answered evasively, downplaying the risks. This aligns with previous research, showing that pharmacists use “vague, verbal descriptions of frequency–rather than numerical indications.”^55^ Customers are more likely to take a medication if the associated risks are framed positively.^55^ Presenting information negatively may increase the risk of losing a sale, which the employee might seek to avoid.^29^ Because understanding is vital for correctly using medications, pharmacy staff must develop new strategies to introduce and clarify information for NPPs.

Pharmacies classified as moderately busy or busy were more likely to perform assessments and give information. This is surprising, as time constraints are frequently cited as a significant challenge in the community pharmacy sector.^56^, 57., 58 The tasks performed by the employees when not counseling customers were not investigated in this study. However, it was observed that the busier pharmacies had more personnel available. A study in a hospital dispensary pharmacy setting revealed that pharmacists spent most of their time not communicating with customers and only 5.8 % of their time in the self-care section. Pharmacy technicians spent more time in the self-care section.^59^ This is also reflected in the present study, where pharmacy technicians typically positioned themselves in the self-care section. Pharmacists in this study predominantly remained behind the prescription counter, venturing into the self-care section only when necessary. This task distribution may be attributed to pharmacists' presence being mandatory for all prescription transactions. However, it might also contribute to fostering the perception that OTC medications are relatively less harmful than prescription-only medications,^14^^,^^15^ not only among the public but also within the profession. Previous studies have mixed findings on education levels and standard compliance.^16^^,^^32^^,^^35^, ^36^, ^37^^,^^60^^,^^61^ Nevertheless, a systematic review of the pharmacist's role in OTC consultations showed that pharmacists conduct better consultations than pharmacy technicians.^62^ As such, the higher odds of standard compliance among pharmacists in this study when counseling on OTCs indicate their expertise is needed in the self-care section.

### 5.1 Implications for practice

Overall, the employees in this study showed inadequate adherence to the Standard's requirements.^22^ However, the Standard merely outlines the necessary tasks without specifying the methods for accomplishing them, and the results indicate that it may be an imperfect instrument for guiding daily operational practices. Considering the results of this study, it would be advisable to revise the current Standard in the future. However, as other studies have shown, more detailed standards do not necessarily mean that employees have better standard compliance.^27^^,^^31^^,^^32^^,^^37^ Still, we argue that the first step would be to design a standard that possibly includes NPPs other than OTCs. This framework could enable and support continuous quality development in the field. Emphasis should be placed on the factors that positively influence counseling, such as engaging with the customer before product selection, utilizing pharmacist resources better in OTC encounters, and addressing topics other than practical use. This responsibility should be shared between owners, managers, and employees. An increased use of checklists for select indications may help ensure equal counseling in each encounter.^63^ However, research on the use of a checklist for non-prescription sildenafil has shown little compliance with this checklist.^64^ Due to the many indications and products available for self-care, careful consideration should be taken when designing such checklists, and feasibility studies should be conducted to assess which NPP sales would benefit from a checklist approach. Increasing focus on communication skills and how to address all customers can help meet self-care goals and alleviate strain in the healthcare sector. This focus should be implemented both during pharmaceutical education and in pharmacy practice. The possibility of recertification programs focusing on communication skills should be explored.^65^^,^^66^ Implementing audits for continuous evaluation of the pharmacy practice quality, with feedback on performance to the employees,^67^ may help increase their awareness of their counseling habits. Further, encouraging customers to obtain more information in the pharmacies, for instance, through question prompt lists, could increase the customers' engagement in non-prescription encounters.^50^ All these efforts must consider the working conditions of the pharmacy employees and involve both employees and other stakeholders to be efficient. Further research on the employees' and the customers' views is needed, especially to understand the motivators of good counseling and the hindrances when meeting uninterested customers.

### 5.2 Strengths and limitations

Non-participant observations offer a genuine view of pharmacy operations and customer interactions without creating an artificial environment.^43^ They provide insight into everyday pharmacy work with actual customers. It can be argued that the observations in this study were not truly non-participatory, as the observers interacted with both customers and employees.^43^ The reactive (Hawthorne) effect, where individuals being observed demonstrate improved performance due to awareness of the observation, might pose a limitation when using this observation method.^43^ In most encounters, no assessment was made, nor was information provided similar to what Seiberth et al. found in their research.^27^ Even though the observers were known, the findings indicate that the majority of customer interactions did not meet the Standard, suggesting that the baseline performance might be even lower.

Audio recordings might have ensured an accurate recall of the conversations' content, but recruitment and recording quality difficulties prevented their use beyond the initial pilot. The observers experienced that customers were reluctant to participate if they were audio recorded. Because the equipment only allowed one portable microphone and the design required mobility, recording both parts of the conversation was challenging. Further, there was a risk of recording other customers who had not consented to being recorded. Instead, two observers acted independently and discussed their notes immediately after each encounter to improve accuracy. The goal of the discussion after the observations was to reach a consensus on the content of the conversation. Observer HG wrote down as much of the conversation as possible, and even though written notes cannot be replicated in the same way as audio recordings, the transcripts were as close to verbatim as possible. Nevertheless, the transcripts cannot be used for a detailed analysis of the language used in the conversations. Still, they are accurate enough for the scope of this research. The study was designed as a qualitative observational study, using limited pre-defined checkboxes to assess the encounters. This provides rich data that describe the encounters and aspects that may not have been predicted before the observations. Codes were discussed among 4 researchers, increasing the validity of the results. Content analysis often uses a preset codebook and multiple coders to improve reproducibility and validity.^47^ The evaluation of compliance with the various requirements of the Standard was conducted through qualitative judgment of the material. Outcome 3 was assessed based on whether the employee provided more extensive information and asked questions to evaluate the customer's condition. It is important to note that customers were not asked whether the information provided met their needs. This evaluation may have been subject to researcher bias, representing a potential study limitation. Nevertheless, the research team thoroughly discussed and reached a consensus on the evaluation of this criterion.

Another limitation of the study is that the outcomes were measured on a dichotomous scale, and there was no differentiation between different degrees of quality in the outcomes. This was done because the Standard does not differentiate between minimum and maximum levels of counseling. Developing evaluation tools for the levels of counseling would be beneficial for further research.

In observational research, the sample size tends to be smaller than that in extensive quantitative studies, precluding definite conclusions on uniform practices. Recruitment was done in a limited geographical area. Nevertheless, this study included various pharmacies and all major pharmacy chains in Norway. The alignment of this study's results with comparable studies^27^^,^^37^ may indicate that the results represent general pharmacy practices. Furthermore, all data were exploratory, so the sample was limited. However, the sample was large enough, for example, the statistical power was sufficient to fit several multiple regression models. Given the sample size limitations, only up to 9 variables were included in the statistical models. The variables needed to have at least 10 encounters to be considered for the model, and variables were evaluated to be able to show statistical power. Due to low numbers of observations for some variables, it was not possible to estimate the effect of some of the selected factors (for assessment: no specific product request; for tailored information: assessment yes, other medical products, age group, and busy pharmacy) with sufficient precision, as is reflected in the extensive confidence intervals of the odds ratios. However, these factors were still highly statistically significant and associated with increased standard compliance, even though their effect size could not be estimated. Sub-analysis, for instance, on only pharmacists, could have improved the understanding of the pharmacist's professional role. The statistical power would be too limited to conduct such an analysis.

## 6 Conclusion

This study shows that employees provide counseling to varying degrees and often do not fully comply with the Norwegian standard for OTC counseling. Factors influencing standard compliance are the employees being available to the customer and interacting with the customer before product selection. The chances of standard compliance are higher for other NPPs than OTCs. Further efforts are needed to improve the counseling on all NPPs. This should consist of revising the standard, drawing on experiences from this and previous studies, and focusing on continuous communication skill promotion among pharmacy employees.

## CRediT authorship contribution statement

**Helene Marie Haldorsen Gombos:** Writing – original draft, Visualization, Project administration, Methodology, Investigation, Formal analysis, Data curation, Conceptualization. **Tonje Krogstad:** Writing – review & editing, Validation, Supervision, Project administration, Methodology, Investigation, Funding acquisition, Conceptualization. **Marthe Rambøl Bjørknes:** Writing – review & editing, Validation, Investigation. **Hege Sletvold:** Writing – review & editing, Validation, Supervision, Methodology, Funding acquisition, Conceptualization. **Milada Cvancarova Hagen:** Writing – review & editing, Resources, Formal analysis. **Karin Svensberg:** Writing – review & editing, Validation, Supervision, Methodology, Conceptualization.

## Declaration of generative AI and AI-assisted technologies in the writing process

During the preparation of this work, the authors used ChatGPT 4.0 and SIKT GPT 4.0 in order to improve language and readability. After using this tool, the authors reviewed and edited the content as needed. They take full responsibility for the content of the publication.

## Funding

This research was supported by Stiftelsen til Fremme av Norsk Apotekfarmasi (The Foundation for the Advancement of Norwegian Community Pharmacy) [reference 2021/07].

## Declaration of competing interest

The authors declare that they have no known competing financial interests or personal relationships that could have appeared to influence the work reported in this paper.

## Appendix A Supplementary data

Supplementary material 1 Image 1

Supplementary material 2 Image 2

Supplementary material 3 Image 3

Supplementary material 4 Image 4

Supplementary material 5 Image 5

## References

[1] Hanlon J.T., Fillenbaum G.G., Ruby C.M., Gray S., Bohannon A. (2001). Epidemiology of over-the-counter drug use in community dwelling elderly: United States perspective. Drugs Aging.

[2] Rogge M., Kumar P., Grundmann O., Committee A.P.P. (2022). Front-line health care professionals lack critical knowledge in dietary supplement and nutraceutical products: a call to action for comprehensive educational opportunities. J Clin Pharmacol.

[3] Association of the European Self-Care Industry (2022). AESGP Annual Report 2022. https://aesgp.eu/cms/annual-report-2022/.

[4] World Health Organization (1998). Report of the 4th WHO Consultative Group on the Role of the Pharmacist. The Role of the Pharmacist in Self-Care and Self-Medication. https://iris.who.int/bitstream/handle/10665/65860/WHO_DAP_98.13.pdf?sequence=1&isAllowed=y.

[5] World Health Organization, International Pharmaceutical Federation (FIP) Joint FIP/WHO Guidelines on Good Pharmacy Practice: Standards for Quality of Pharmacy Services. 2011. Report No.: 961. https://www.who.int/docs/default-source/medicines/norms-and-standards/guidelines/distribution/trs961-annex8-fipwhoguidelinesgoodpharmacypractice.pdf.

[6] Rutter P., Barnes N. (2024). Facilitating self-care through community pharmacy in England. Explor Res Clin Soc Pharm.

[7] Bertsche T., Alexa J.M., Eickhoff C., Schulz M. (2023). Self-care and self-medication as central components of healthcare in Germany – on the way to evidence-based pharmacy. Explor Res Clin Soc Pharm.

[8] Nakhla N., Taylor J. (2024). Self-care and minor ailments: the view from Canada. Explor Res Clin Soc Pharm.

[9] Amador-Fernández N., Jenkinson S.P., Berger J. (2023). Vision and practice of self-care for community pharmacy in Switzerland. Explor Res Clin Soc Pharm.

[10] Westerlund L.T., Marklund B.R., Handl W.H., Thunberg M.E., Allebeck P. (2001). Nonprescription drug-related problems and pharmacy interventions. Ann Pharmacother.

[11] Eickhoff C., Hämmerlein A., Griese N., Schulz M. (2012). Nature and frequency of drug-related problems in self-medication (over-the-counter drugs) in daily community pharmacy practice in Germany. Pharmacoepidemiol Drug Saf.

[12] Bennadi D. (2013). Self-medication: a current challenge. J Basic Clin Pharm.

[13] Ylä-Rautio H., Siissalo S., Leikola S. (2020). Drug-related problems and pharmacy interventions in non-prescription medication, with a focus on high-risk over-the-counter medications. Int Clin Pharm.

[14] Cherkasova A.V., Kuryta A.V., Lysunets T.K. (2014). NSAIDs: patient’s beliefs and perceptions. What is the REAL situation?. Ann Rheum Dis.

[15] Paliwal Y., Gendron T.L., Jones R.M., Moczygemba L., Nadpara P.A., Slattum P.W. (2019). A qualitative study to understand over-the-counter medication use and decision-making among residents of senior-living communities. Res Soc Adm Pharm.

[16] Emmerton L., Shaw J. (2002). The influence of pharmacy staff in non-prescription medicine sales. Int J Pharm Pract.

[17] Volmer D., Lilja J., Hamilton D. (2007). How well informed are pharmacy customers in Estonia about minor illnesses and over-the-counter medicines. Medicina (Kaunas).

[18] Smailhodzic E., Hooijsma W., Boonstra A., Langley D.J. (2016). Social media use in healthcare: a systematic review of effects on patients and on their relationship with healthcare professionals. BMC Health Serv Res.

[19] Hughes L., Whittlesea C., Luscombe D. (2002). Patients’ knowledge and perceptions of the side-effects of OTC medication. J Clin Pharm Ther.

[20] Bergmo T.S., Sandsdalen V., Manskow U.S., Smabrekke L., Waaseth M. (2023). Internet use for obtaining medicine information: cross-sectional survey. JMIR Form Res.

[21] Forskrift om Rekvirering og Utlevering av Legemidler m.m. [Regulation on the Prescribing and Dispensing of Medicinal Products, etc.], FOR-2022-06-02-977 (2022). https://lovdata.no/dokument/SF/forskrift/2022-06-02-977. (Access date 03.01.2025) [Norwegian]..

[22] Apotekforeningen. Rådgivning Ved Salg av Reseptfrie Legemidler [Counseling During the Sale of OTC Medications]. 2016. https://www.apotek.no/Files/Filer_2014/Apotekbransjen/bransjestandard%20reseptfrie.pdf. [Norwegian].

[23] ASHP (1997). ASHP guidelines on pharmacist-conducted patient education and counseling. Am J Health Syst Pharm.

[24] Federal Chamber of Pharmacists. Leitlinie der Bundesapothekerkammer. zur Qualitätssicherung [Guideline: Information and Counselling of Patients in the Supply of Medicines – Self-Medication]. 2023. https://www.abda.de/fileadmin/user_upload/assets/Praktische_Hilfen/Leitlinien/Selbstmedikation/LL_Info_Beratung_SM.pdf. [German].

[25] Pharmaceutical Society of Australia (2023). Professional Practice Standards 2023 Version 6. https://www.psa.org.au/practice-support-industry/pps/.

[26] Watson M.C., Bond C.M., Johnston M., Mearns K. (2006). Using human error theory to explore the supply of non-prescription medicines from community pharmacies. Qual Saf Health Care.

[27] Seiberth J.M., Moritz K., Herrmann N.S., Bertsche T., Schiek S. (2022). What influences the information exchange during self-medication consultations in community pharmacies? A non-participant observation study. Res Soc Adm Pharm.

[28] Watson M.C., Hart J., Johnston M., Bond C.M. (2008). Exploring the supply of non-prescription medicines from community pharmacies in Scotland. Pharm World Sci.

[29] Seiberth J.M., Moritz K., Kücükay N., Schiek S., Bertsche T. (2020). What is the attitude towards and the current practice of information exchange during self-medication counselling in German community pharmacies? An assessment through self-report and non-participant observation. PLoS One.

[30] Langer B., Kunow C. (2022). The quality of counseling for headache OTC medications in German community pharmacies using a simulated patient approach: are there differences between self-purchase and purchase for a third party?. Sci World J.

[31] Smith H., Whyte S., Chan H.F. (2019). Pharmacist compliance with therapeutic guidelines on diagnosis and treatment provision. JAMA Netw Open.

[32] Minarikova D., Fazekas T., Minarik P., Jurisova E. (2019). Assessment of patient counselling on the common cold treatment at Slovak community pharmacies using mystery shopping. Saudi Pharm J.

[33] Veiga P., Lapão L.V., Cavaco A.M., Guerreiro M.P. (2015). Quality supply of nonprescription medicines in Portuguese community pharmacy: an exploratory case study. Res Soc Adm Pharm.

[34] Schneider C.R., Emery L., Brostek R., Clifford R.M. (2013). Evaluation of the supply of antifungal medication for the treatment of vaginal thrush in the community pharmacy setting: a randomized controlled trial. Pharm Pract (Granada).

[35] Horvat N., Koder M., Kos M. (2012). Using the simulated patient methodology to assess paracetamol-related counselling for headache. PLoS ONE.

[36] Alte D., Weitschies W., Ritter C.A. (2007). Evaluation of consultation in community pharmacies with mystery shoppers. Ann Pharmacother.

[37] Watson M.C., Bond C.M., Grimshaw J., Johnston M. (2006). Factors predicting the guideline compliant supply (or non-supply) of non-prescription medicines in the community pharmacy setting. Qual Saf Health Care.

[38] Alastalo N., Siitonen P., Jyrkkä J., Hämeen-Anttila K. (2023). The quality of non-prescription medicine counselling in Finnish pharmacies – a simulated patient study. Explor Res Clin Soc Pharm.

[39] El-Souri M., Hansen R.N., Raagaard A.M., Sondergaard B., Rossing C. (2020). Pharmacy technicians’ contribution to counselling at community pharmacies in Denmark. Pharmacy.

[40] Seubert L.J., Whitelaw K., Boeni F., Hattingh L., Watson M.C., Clifford R.M. (2017). Barriers and facilitators for information exchange during over-the-counter consultations in community pharmacy: a focus group study. Pharmacy.

[41] Moritz K., Seiberth J.M., Herrmann N.S., Bertsche T., Schiek S. (2021). Are evidence-based criteria addressed during counseling on over-the-counter products? An observational study in community pharmacies. Patient Educ Couns.

[42] Seiberth J.M., Moritz K., Vogel C.F., Bertsche T., Schiek S. (2021). Public’s perspectives on guideline-recommended self-medication consultations in German community pharmacies. Health Soc Care Commun.

[43] Johannessen A. (2016).

[44] O’Brien B.C., Harris I.B., Beckman T.J., Reed D.A., Cook D.A. (2014). Standards for reporting qualitative research: a synthesis of recommendations. Acad Med.

[45] (2003). Forskrift om omsetning mv. av Visse Reseptfrie Legemidler Utenom Apotek [Regulation on the Sales of Certain Non-Prescription Medications Outside Pharmacies], FOR-2023-01-16-50. https://lovdata.no/forskrift/2003-08-14-1053.

[46] (1999). Lov om Helsepersonell m.v., LOV-1999-07-02-64. https://lovdata.no/dokument/NL/lov/1999-07-02-64.

[47] Krippendorff K. (2019).

[48] (2008). Lov om Medisinsk og Helsefaglig Forskning (LOV-2008-06-20-44). https://lovdata.no/dokument/NL/lov/2008-06-20-44/KAPITTEL_3#KAPITTEL_3.

[49] Langer B., Kunow C., Bolduan J. (2024). Counselling with a focus on product and price transparency for over-the-counter headache medicines: a simulated patient study in community pharmacies in Munich, Germany. Int J Health Plann Manag.

[50] Svensberg K., Kaae S., Mottelson N.B., Persson C.L. (2024). Identifying critical elements in using question prompt lists at the pharmacy counter to induce patient activation—using principles of conversation analysis. Res Soc Adm Pharm.

[51] Kaune A., Schumacher P.M., Hoppe S.C. (2016). Administration of anticonvulsive rescue medication in children—discrepancies between parents’ self-reports and limited practical performance. Eur J Pediatr.

[52] Mira J.J., Lorenzo S., Guilabert M., Navarro I., Pérez-Jover V. (2015). A systematic review of patient medication error on self-administering medication at home. Expert Opin Drug Saf.

[53] Puspitasari H.P., Aslani P., Krass I. (2009). A review of counseling practices on prescription medicines in community pharmacies. Res Soc Adm Pharm.

[54] Twigg M.J., Bhattacharya D., Clark A. (2016). What do patients need to know? A study to assess patients’ satisfaction with information about medicines. Int J Pharm Pract.

[55] Dyck A., Deschamps M., Taylor J. (2005). Pharmacists’ discussions of medication side effects: a descriptive study. Patient Educ Couns.

[56] Thorsted C.K. (2023). Apoteksfarmaceuters psykosociale arbejdsmiljø: brug for handling [Community pharmacists psykosocial work environment: call for action]. Pharmadanmark.

[57] Bednall R. (2019). Why are pharmacists feeling under pressure at work?. Rx Clinical Pharm Mag.

[58] Conelly D. (2021). Work-related stress: the hidden pandemic in pharmacy. Pharm J.

[59] Lehnbom E.C., Rakicevic A., Skjold F., Svendsen K., Andersson Y., Elenjord R. (2024). A time and motion study in two Norwegian hospital dispensary pharmacies: a direct observational study of pharmacists and pharmacy technicians. Norsk Farmac Tidskrift.

[60] Langer B., Kunow C. (2019). Do north-eastern German pharmacies recommend a necessary medical consultation for acute diarrhoea? Magnitude and determinants using a simulated patient approach. F1000Res.

[61] Kashyap K.C., Nissen L.M., Smith S.S., Kyle G. (2014). Management of over-the-counter insomnia complaints in Australian community pharmacies: a standardized patient study. Int J Pharm Pract.

[62] van Eikenhorst L., Salema N.E., Anderson C. (2017). A systematic review in select countries of the role of the pharmacist in consultations and sales of non-prescription medicines in community pharmacy. Res Soc Adm Pharm.

[63] Langer B., Grimm S., Lungfiel G., Mandlmeier F., Wenig V. (2020). The quality of counselling for oral emergency contraceptive pills—a simulated patient study in German community pharmacies. Int J Environ Res Public Health.

[64] Syversen H.T., Krogstad T., Sletvold H. (2024). Pharmacist supply of non-prescription sildenafil in Norway: a simulated patient mixed-method study. Int J Pharm Pract.

[65] Potter H., Hassell K., Noyce P.R. (2013). Pharmacists’ and pharmacy technicians’ views on a process of revalidation of pharmacy professionals in Great Britain. Res Soc Adm Pharm.

[66] Schafheutle E.I., Hassell K., Noyce P.R. (2013). Ensuring continuing fitness to practice in the pharmacy workforce: understanding the challenges of revalidation. Res Soc Adm Pharm.

[67] Collins J.C., Schneider C.R., Naughtin C.L., Wilson F., de Almeida Neto A.C., Moles R.J. (2017). Mystery shopping and coaching as a form of audit and feedback to improve community pharmacy management of non-prescription medicine requests: an intervention study. BMJ Open.

