# Supplementary material for: A qualitative non-participant observational study of non-prescription counseling in community pharmacies
Source: Explor Res Clin Soc Pharm. 2025 May 3;18:100611. doi: 10.1016/j.rcsop.2025.100611 (PMC12146651; doi:10.1016/j.rcsop.2025.100611)
Supplement: Supplementary file 1 — Supplementary material 1 [file mmc1.pdf]

Observation number: **Pharmacy number:** **Employee number:**

Age group:
- ☐ 18-49
- ☐ 50-69
- ☐ 70+

Gender:
- ☐ Male
- ☐ Female
- ☐ Neutral

Date:
Time:
How long:

Product:
Name | Form of administration | Doseage

☐ No product

Other customers
Other employees
Employee:
- ☐ Restocks
- ☐ Waits
- ☐ Reads
- ☐ Talking on phone
- ☐ In prescription area
- ☐ Helping other customer

Start of conversation
- ☐ Employee makes contact
- ☐ Customer makes contact

Comments:

Non-verbal communication
- ☐ Eye contact
- ☐ Open body language
- ☐ Active listening
- ☐ Nods
- ☐ Smiles
- ☐ Show Empathy
- ☐ Acknowledges
- ☐ Appropriate use of breaks
- ☐ Appropriate questioning

Notes from conversation

Observation number: **Pharmacy number**: **Employee number**:

Age group:
- ☐ 18-49
- ☐ 50-69
- ☐ 70+

Gender:
- ☐ Male
- ☐ Female
- ☐ Neutral

Date:
Time:
How long:

Product:
Name Form of administration Doseage

☐No product

Other customers
Other employees
Employee:
- ☐ Restocks
- ☐ Waits
- ☐ Reads
- ☐ Talking on phone
- ☐ In prescription area
- ☐ Helping other customer
- ☐ Other_____

Non-verbal communication
- ☐ Eye contact
- ☐ Open body language
- ☐ Active listening
- ☐ Nods
- ☐ Smiles
- ☐ Show Empathy
- ☐ Acknowledges
- ☐ Appropriate use of breaks
- ☐ Appropriate questioning

Start of conversation
- ☐ Employee makes contact
- ☐ Customer makes contact

Comments:

Who is the patient?

What are the symptoms?

When did the symptoms start?

What has been done previously?

Topic	Yes	No	Not asked	Comments. NB! Justification
Use other medications				
Comorbidities				
Allergy/intolerence				

Customers previous experience

Employees previous experience

Documented effect

Topic	Yes	No	Uncomplete	Comments. NB! Justification
Dosage				
Clinical effect				
Adverse drug reaction				
Administration				
Length of treatment				
Instructions				
Contraindications				
Refer to physician				
Written info/insert				
Appropriate ending				
Professionally secure				
