# Supplementary material for: A qualitative non-participant observational study of non-prescription counseling in community pharmacies
Source: Explor Res Clin Soc Pharm. 2025 May 3;18:100611. doi: 10.1016/j.rcsop.2025.100611 (PMC12146651; doi:10.1016/j.rcsop.2025.100611)
Supplement: Supplementary file 2 — Supplementary material 2 [file mmc2.docx]

## Codebook sample

| Codes | Description |
| --- | --- |
| Assessments |  |
| Age of user | The age for which the product is intended, or the age of the patient who will receive the product |
| Assessing practical needs | Employee asks questions to find out what the customer needs, to be able to recommend product or give advice |
| Breastfeeding | Asking about breastfeeding |
| Comorbidities | Conditions that may cause the symptoms, and not contraindications for using the medicine |
| Contraindications | Employee asks about comorbidities, may explain why question is asked |
| dietary questions | Questions/information about diet to assess what to recommend |
| Duration of symptoms | Employee asks about, or the customer otherwise tells, how long the symptoms have occurred |
| frequency of symptoms | How often does the customer have the symptoms |
| Product selection verification | Customer has found a product, the employee makes sure that the customer found the right product, and corrects it if not |
| Interactions | Asks about other medicines, sometimes giving advice about medicines together, or just assessing if the customer can safely use the medication |
| Other products used previously | Employee asks, or the customer says, if they have used any other products, medicines etc. for the same ailments previously. Sometimes this can mean recently, others it may seem like the customer have used it at a different time |
| Pregnancy | Employee asks about pregnancy |
| Previous contact with physician | Employee asks if the customer has consulted with a doctor previously, or the customer says that they have been to the doctor, and they recommended this treatment |
| Previous experience | The customer has used the product previously, or says they have previous knowledge of the product |
| Symptoms | The employee asks about symptoms to be able to recommend a product. Part of assessing needs, but more specific |
| Treating adverse drug reactions of other medicines | The customer says the use other medication, and need something to treat side effects of this treatment |
| Who is the product for | The employee asks who the product is for, or the customer says themselves that the product is for someone else |
| Conversation at point of sales | Conversation starts at the point of sales |
| Conversation at shelf | Conversation starts at the shelf |
| Finding product |  |
| Customer asks for specific OTC product | Customer does not find the product themselves, but asks the employee for a specific, named OTC product |
| customer asks for specific product | Customer does not find the product themselves, but asks the employee for a specific, named product |
| Customer asks for unspecific OTC product | Customer presents a symptom, or product group, wishing for the employees input on a specific product, or being pointed in the right direction to find the product they need |
| Customer asks for unspecific product | Customer presents a symptom, or product group, wishing for the employees input on a specific product, or being pointed in the right direction to find the product they need |
| Customer finds OTC product themselves | Customer picks OTC product from the shelf, without consulting with an employee beforehand |
| Customer finds product themselves | Customer picks product from the shelf, without consulting with an employee beforehand |
| Information |  |
| Administration | How should the product be administered |
| Adverse drug reactions | Information on adverse drug reactions is provided |
| Area of application | When employee describes where a product should be used, topically, orally, nasally etc. |
| Dependency | The customer asks if it is possible to be dependent on the OTC medicine |
| Dietary information | Employee gives information about dietary recommendations, including liquid intake, when using a product |
| Dose on package or insert | Employee explains that the dosing is written on the package or the insert, but does not say the dose to the customer |
| Dosage quantity and frequency | The employee states, or explains, how and when product should be dosed |
| Clinical effect | Employee explains the effect of a product, without the customer asking about the effect |
| Expiration date | Talking about expiration of products |
| Formulation | Giving options of different forms of medicine, or telling what form the medicine is in |
| Generic substitution | The customer finds an OTC product, and the employee suggest a different, cheaper product |
| Indication | The employee asks or says what the product is used against/for |
| Contraindication | Employee does not ask about contraindications, but gives information about diseases where the product is contraindicated |
| Interactions | Employee does not ask if a customer uses other medicines, but gives information about medications that should not be used with purchased product |
| Practical instruction of use | Practical instructions for use of product |
| Length of treatment | The employee stated how long a product should or can be used |
| Long term use | Employee addresses issues with long term use of a product |
| Only mentioning maximum total dosage | Employee states what the maximum daily dose is, without giving details of each dose, or how often |
| Offering measuring cup | Employee offers a tool (e.g. measuring cup, syringe) or makes sure customer has what is needed to administer product |
| Only mentioning “Follow the dosage” | Saying that the customer should be careful with the dosing, without saying where the dose can be found, or the actual dose |
| Non-medical advice | Employee gives advice that is not strictly medical (use of medicine) but can help with symptoms |
| Other alternative treatments | Employee talking about other products that can be used if no effect etc. |
| Miscellaneous recommendations | Advice that does not fit other categories, e.g. prunes for constipation |
| Complimentary treatment | Employee recommends additional treatment with non-medical products, complimentary to the medical treatment |
| Sales restrictions | There is a limit to how much of the product that can be sold at once, and the employee addresses this issue |
| Storage | Employee gives information about how to store a product |
| Concurrent medication | Giving information about medicines that can be used together |
| Written information given | Employee provides written information |
| Information classification |  |
| Dose, little other information | Employee states the dose, gives little other information in the encounter. |
| More information given | When the employee gives more information than just generic |
| No OTC information given | Employee sells an OTC-medicine without giving any information. Does not apply for supplements etc. |
| Generic information with dose | Employee gives standardized information, such as dosing on package, limited use, information of contraindication without asking if this applies to the customer. |
| Generic information without dose | Some generic information is provided, but dosage is not given |
| Type of sales |  |
| Medical technical product sold | product classified as medical technical product is sold. |
| No OTC product sold | Encounters without sale of OTC-products. |
| No sale | When there is nothing sold in the encounter, either because the employee recommends talking with the doctor, just gives advice, or because the pharmacy is sold out. |
| OTC product sold | sale of OTC medicine in encounter. |
| supplement sold | supplements sold in the encounter |
