# Supplementary material for: A qualitative non-participant observational study of non-prescription counseling in community pharmacies
Source: Explor Res Clin Soc Pharm. 2025 May 3;18:100611. doi: 10.1016/j.rcsop.2025.100611 (PMC12146651; doi:10.1016/j.rcsop.2025.100611)
Supplement: Supplementary file 3 — Supplementary material 3 [file mmc3.docx]

| **Table S1:** Factors related to nformation provided unsolicited about OTC | | | |
| --- | --- | --- | --- |
| **Variables** | **OR** | **95% CI** | **p-value** |
| **Assessment** |  |  |  |
| *No (reference)* | 1 |  |  |
| *Yes* | 3.10 | 1.22 – 10.01 | <0.001 |
| **Location in pharmacy** |  |  |  |
| *At shelf (reference)* | 1 |  |  |
| *At point of sales* | 2.56 | 0.68 – 24.41 | 0.049 |
| **Employee education** |  |  |  |
| *Pharmacist (reference)* | 1 |  |  |
| *Pharmacy technician* | 0.386 | 0.29 – 0.79 | 0.010 |
| **Pharmacy busyness** |  |  |  |
| *Quiet (reference)* | 1 |  |  |
| *Medium busy* | 2.40 | 1.00 – 6.78 | 0.033 |
| *Busy* | 1.72 | 1.63 – 4.84 | 0.232 |

| **Table S2:** Factors related to assessment of customer´s OTC product needs | | | |
| --- | --- | --- | --- |
| **Variables** | **OR** | **95% CI** | **p-value** |
| **Specific product** |  |  |  |
| *Yes (reference)* | 1 |  |  |
| *No* | 15.99 | 3.34 - * | <0.001 |
| **Previous physician contact** |  |  |  |
| *No (reference)* | 1 |  |  |
| *Yes* | 3.64 | 1.22 – 15.07 | 0.015 |
| **Pharmacy busyness** |  |  |  |
| *Quiet (reference)* | 1 |  |  |
| *Medium busy* | 4.00 | 5.52 - * | 0.006 |
| *Busy* | 1.81 | 1.90 – 167.17 | 0.272 |
| **Location in pharmacy** |  |  |  |
| *At point of sales (reference)* | 1 |  |  |
| *At shelf* | 6.80 | 2.35 – 66.42 | <0.001 |

* could not be estimated

| **Table S3:** Factors related to assessment of customer´s information needs about OTC | | | |
| --- | --- | --- | --- |
| **Variables** | **OR** | **95% CI** | **p-value** |
| **Employee education** |  |  |  |
| *Pharmacist* | 1 |  |  |
| *Pharmacy technician* | 0.805 | 0.26 – 2.26 | 0.695 |
| **Location in pharmacy** |  |  |  |
| *At point of sales (reference)* | 1 |  |  |
| *At shelf* | 9.975 | 2.30 – 69.69 | <0.001 |
