# Supplementary material for: A qualitative non-participant observational study of non-prescription counseling in community pharmacies
Source: Explor Res Clin Soc Pharm. 2025 May 3;18:100611. doi: 10.1016/j.rcsop.2025.100611 (PMC12146651; doi:10.1016/j.rcsop.2025.100611)
Supplement: Supplementary file 4 — Supplementary material 4 [file mmc4.docx]

Supplementary File 2a


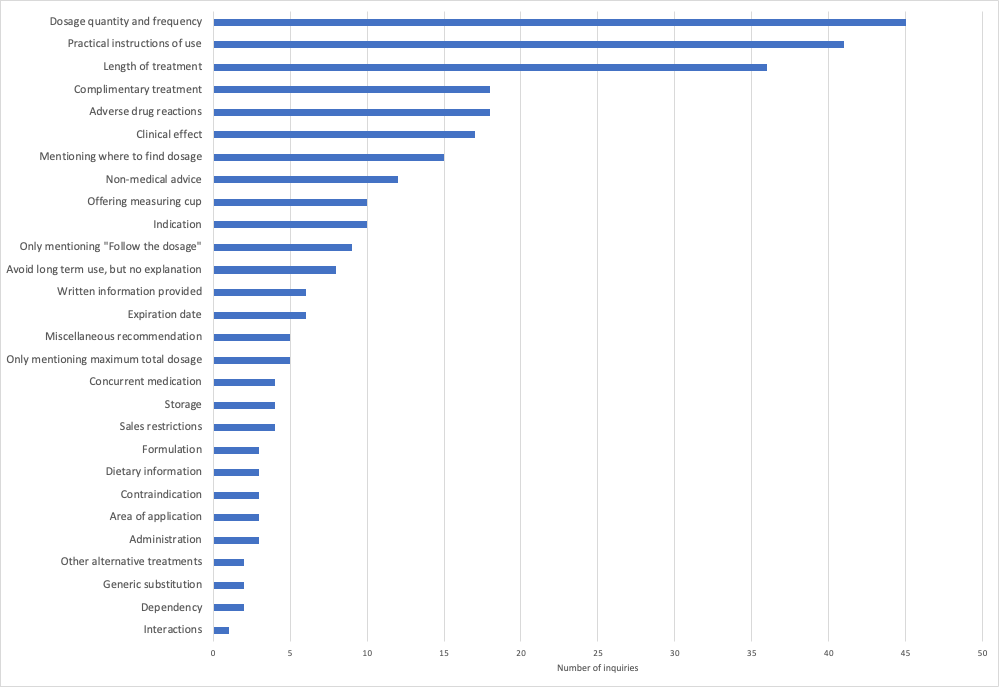


*Type of information given during counseling customers in non-prescription product encounters.*

Supplementary File 2b


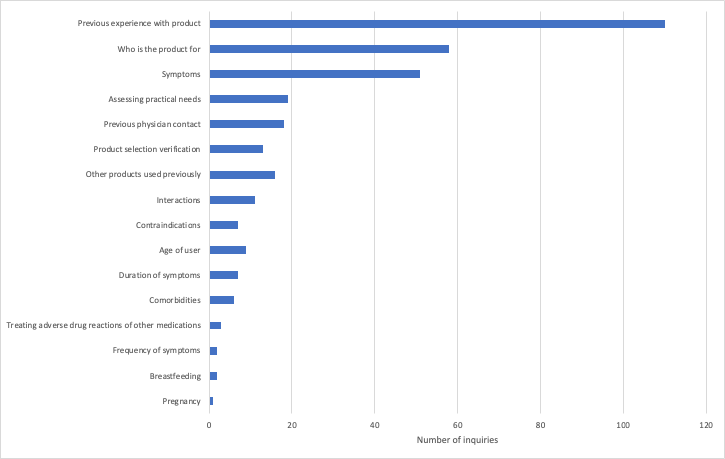


*Topics discussed during the assessment of customers in non-prescription product inquiries.*
